# “More crop per drop”: Exploring India's cereal water use since 2005

**DOI:** 10.1016/j.scitotenv.2019.03.304

**Published:** 2019-07-10

**Authors:** Benjamin Kayatz, Francesca Harris, Jon Hillier, Tapan Adhya, Carole Dalin, Dali Nayak, Rosemary F. Green, Pete Smith, Alan D. Dangour

**Affiliations:** a University of Aberdeen, Aberdeen, UK; b Helmholtz Centre Potsdam German Research Centre for Geosciences, Potsdam, Germany; c London School of Hygiene & Tropical Medicine, London, UK; d Global Academy of Agriculture and Food Security, The Royal (Dick) School of Veterinary Studies and The Roslin Institute, Easter Bush Campus, Midlothian, UK; e Kalinga Institute of Industrial Technology, Bhubaneswar, India; f Institute for Sustainable Resources, University College London, UK; g Brandenburg University of Technology Cottbus-Senftenberg, Cottbus, Germany

**Keywords:** Cereals, India, Water resource management, Food security, Irrigation, Cool Farm Tool

## Abstract

India has the highest national freshwater demand globally and 91% of India's freshwater is used in the agriculture sector. Cereals account for over 50% of the dietary water footprint in India and represent a potential opportunity for reducing water use in Indian agriculture.

This study combines governmental production and irrigation statistics with crop distribution maps to examine trends in annual water use for cereal production in India between 2005 and 2014. A new online water assessment tool, Cool Farm Tool Water (CFTW), was used to calculate water use and derive seasonal state-level blue and green water footprints for rice, wheat, sorghum, millet and maize.

The analysis indicates that India achieved 26.4% increased total cereal production between 2005 and 2014 without additional water or land use. Cereal water footprints have declined due to higher yields for most crops and slightly lower rates of evapotranspiration. There has also been a shift in the area under production away from the *Kharif* (monsoon) towards the *Rabi* (dry) season in which total water footprints for all cereals except rice are substantially lower (−33.4% to −45.0% compared to *Kharif*), but show a significantly higher dependency on ground and surface water.

The value of this study is two-fold. First, it provides a full assessment of production trends for the five major cereals in India for each year from 2005 to 2014 and links it to water use. Secondly, it uses updated seasonal water footprints, which demonstrate the potential for changes in cereal production practices to contribute to improved efficiency of water use in India. Future pressures on scarce water resources may encourage transition to cereals with lower irrigation dependency, in particular maize, but also sorghum and millet. In addition, increased emphasis on improving millet and sorghum yields would be of benefit to secure cereal production and reduce its overall water footprint.

## Highlights

• Between 2005 and 2014 the production of 5 dominant cereals in India increased by 26%.
• Total water use for cereal production in India has remained largely unchanged.
• Cereals are increasingly produced in the dry (Rabi) season.
• Enhancing maize, millet and sorghum production may reduce India's agricultural use of freshwater.
• Millet and sorghum yields are currently too low to ensure sufficient total cereal supply.

## Graphical abstract

**Figure:**
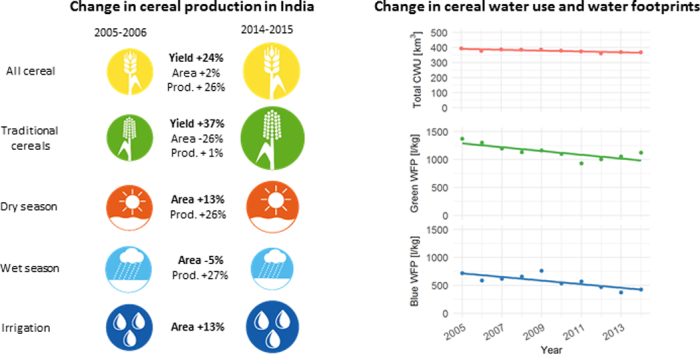

## 1 Introduction

Feeding a growing world population with healthy diets, while minimizing the impacts on the environment, is one of the biggest challenges of the coming decades (Godfray et al., 2010; Springmann et al., 2018). The population of India has risen by over 235% in the last 60 years (Census of India, 2011), and this has coincided with a large increase in food production due to significant improvements in agriculture through India's Green Revolution. Cereal production shifted away from traditional cereals such as millet and sorghum, and towards higher yielding cereals of rice and wheat. Since 1986, the country has been mostly self-sufficient in cereal production, growing over 223.7 million tonnes of cereals annually (average 2005–2014) (DACNET, 2017; Maitra, 1991; Ministry of Agriculture and Farmers Welfare, 2017).

The increase in cereal production over the last decades has had an impact on the local environment, through increased agricultural land area, fertilizer and water use (Barik et al., 2017). India is the largest consumer of freshwater (ground and surface water) globally, 91% of which is withdrawn for food production (FAO, 2016). Groundwater depletion increased by 23% from 2000 to 2010 (Dalin et al., 2017) and is a major concern for cereal production and self-sufficiency (Barik et al., 2017). Cereals play a dominant role in Indian diets and contribute to approximately 50% of the total water used in the agricultural production of food in India (Harris et al., 2017). Coinciding with the shift in production, cereal consumption patterns have changed with more rice and wheat and less coarse cereals such as millet, maize and sorghum being consumed (DeFries et al., 2018). However, the Indian population suffers a large burden of micronutrient deficiencies, and increasing consumption of nutrient-dense coarse cereals has been proposed as a beneficial public health nutrition intervention (DeFries et al., 2015; Rao et al., 2018).

To advance understanding of the relationship between water use and cereals in India, this study explores trends in cereal production, water use and water footprints of five major cereals (rice, wheat, millet, sorghum and maize) from 2005 to 2014. Crop production, area and irrigation statistics for India were combined with novel data generated through the agricultural water assessment tool, Cool Farm Tool Water (CFTW), to quantify total water use of cereal production (Hillier et al., 2011; Kayatz et al., 2019). The variability in water footprints is analysed for all states and seasons to understand the drivers of total water use. Changes to yields and cropping practices are identified to determine important factors for increased cereal production in India.

## 2 Methods

A spatially and temporally explicit dataset of crop production and irrigation area was used to determine cereal water use in India *via* the CFTW model (Kayatz et al., 2019).

### 2.1 Data

All data used for this study are summarized in Table 1 and described below, including the further processing of the data.

**Table 1:** Overview of datasets used for crop production, crop distribution and irrigation.

Dataset	Source	Spatial resolution	Temporal resolution
District area & production	DACNET (2017)	District	Season & year
State area & production	Ministry of Agriculture and Farmers Welfare, 2016, Ministry of Agriculture and Farmers Welfare, 2017, Ministry of Agriculture, 2015, Ministry of Agriculture, 2014, Ministry of Agriculture, 2013, Ministry of Agriculture, 2012, Ministry of Agriculture, 2011, Ministry of Agriculture, 2009, Ministry of Agriculture, 2008, Ministry of Agriculture, 2007	State	Year
State yield	Ministry of Agriculture and Farmers Welfare, 2016, Ministry of Agriculture and Farmers Welfare, 2017, Ministry of Agriculture, 2015, Ministry of Agriculture, 2014, Ministry of Agriculture, 2013, Ministry of Agriculture, 2012, Ministry of Agriculture, 2011, Ministry of Agriculture, 2009, Ministry of Agriculture, 2008, Ministry of Agriculture, 2007	State	Season & year
Area	Zhao and Siebert (2015)	500 m × 500 m	2005
Irrigation fraction	Zhao and Siebert (2015)	500 m × 500 m	2005
Irrigation fraction	Ministry of Agriculture (2015)	State	Year
Crop calendar	Ministry of Agriculture (2015)	State	Season
Crop calendar	Portmann et al. (2010)	State	Season
Crop calendar	Ikisan (2017)	State	Season
Crop calendar	NFSM (2017)	State	Season

#### 2.1.1 Crop area and production

Publicly available data from the Government of India on crop and cereal production were abstracted for the 5 dominant cereals under production (rice, wheat, maize, millet and sorghum). District-wise data on harvested area, production and yield for all seasons were provided by the Directorate of Economics and Statistics, Ministry of Agriculture and Farmer's Welfare (DACNET, 2017). Additionally, state-level data for crop area and production were available from the “Agriculture Statistics at a Glance Year Book” from 2005 to 2014 on an annual basis for the major producing states (Ministry of Agriculture and Farmers Welfare, 2016, Ministry of Agriculture and Farmers Welfare, 2017; Ministry of Agriculture, 2015, Ministry of Agriculture, 2014, Ministry of Agriculture, 2013, Ministry of Agriculture, 2012, Ministry of Agriculture, 2011, Ministry of Agriculture, 2009, Ministry of Agriculture, 2008, Ministry of Agriculture, 2007. At state-level, only yield data for the different seasons was available.

Gaps in data were identified at both district and state-level. The following process was adopted to align both datasets, fill gaps and provide an overview of cereal production for the whole of India, for all growing seasons between 2005 and 2014.

1. Season names were harmonized under; Autumn, Winter, *Kharif* (monsoon), Summer and *Rabi* (dry).
2. Seasonal state-level yields were used to attribute total state production and area to the growing seasons for states with less than three growing seasons.
3. District and state-level area and production data were harmonized to establish a combined state-level dataset as described in Eqs. (1), (2) below
  (1) $$A_{\mathrm{state},i}=\max\left(A_{\mathrm{state},i},\sum A_{\mathrm{dist},i,j}\right)$$
  (2) $$P_{\mathrm{state},i}=\max\left(P_{\mathrm{state},i},\sum P_{\mathrm{dist},i,j}\right)$$
  where *A*_*state*, *i*_ and *P*_*state*, *i*_ is the state-level value for area and production for state *i*, respectively. *A*_*dist*, *i*, *j*_ and *P*_*dist*, *i*, *j*_ are the production of district *j* in state *i*.
4. Missing district-level area and yield statistics were approximated for every year and season based on a linear relationship using existing district and state data. The total gap-filled district area and production within one state was scaled to match state values determined in step 3.
5. Harvested area within each district was allocated using the gridded GEOSHARE crop distribution map for India for each individual crop (Zhao and Siebert, 2015) using a constant yield for each district, year and season.

#### 2.1.2 Crop irrigation area

The Indian “Agriculture Statistics at a Glance” yearbooks provide the irrigation fraction for every cereal in the main producing states and the national average (Ministry of Agriculture and Farmers Welfare, 2016, Ministry of Agriculture and Farmers Welfare, 2017; Ministry of Agriculture, 2015, Ministry of Agriculture, 2014, Ministry of Agriculture, 2013, Ministry of Agriculture, 2012, Ministry of Agriculture, 2011, Ministry of Agriculture, 2009, Ministry of Agriculture, 2008, Ministry of Agriculture, 2007. Irrigation distribution was taken from the gridded GEOSHARE dataset (Zhao and Siebert, 2015), which provides total irrigated area as a fraction of a 500 m by 500 m grid cell, representative for the year 2005. The raster was re-sampled to a 5 arc-min by 5 arc-min grid (approx. 9 km by 9 km). The total irrigated area was distributed across India as follows:

1. The total irrigated area in India and the states where irrigation fractions are available (between 14 and 30 depending on crop and year) were used to determine the irrigation area that has not been allocated to a state *I*_*notallocated*_ (Eq. (3)).
  (3) $$I_{\mathrm{notallocated}}=I_{\mathrm{India}}-\sum I_{\mathrm{state},i}$$
  where *I*_*India*_ is the total irrigated area of a specific year and *I*_*state*, *i*_ is the irrigated area for state *i* where irrigation information is available. *I*_*notallocated*_ varied between 0.0% and 11.2% depending on crop and year. Wheat had the lowest *I*_*notallocated*_ throughout 2005 to 2014 (max. 0.3%).
2. *I*_*notallocated*_ was allocated to the remaining states without annual irrigation fraction data according to the state fraction from the GEOSHARE dataset (Zhao and Siebert, 2015).
3. Irrigation area was then allocated to individual districts in one state based on district irrigation fraction in the GEOSHARE dataset (Zhao and Siebert, 2015). This study assumes that the distribution of irrigated area of the districts within one state remained constant between 2005 and 2014.
4. Irrigation area in each district was primarily allocated to the *Rabi* and Summer growing season. If the irrigation area exceeds the total area of those seasons, irrigation area is allocated to the monsoon driven seasons (Winter, Autumn and *Kharif*) based on their total area fractions.
5. Irrigation within one district was allocated based on cell fractions using the GEOSHARE dataset (Zhao and Siebert, 2015).

#### 2.1.3 Crop calendars

Sowing and harvesting dates for all five cereals were primarily assimilated from Ministry of Agriculture (2015), Portmann et al. (2010), Ikisan (2017) and NFSM (2017). Crop growing seasons are constant for all years and do not reflect annual variability.

### 2.2 Evapotranspiration and water footprints

Actual evapotranspiration *ET*_*a*_ and potential evapotranspiration *ET*_*pot*_ were determined using CFTW (Kayatz et al., 2019). The Cool Farm Tool (CFT) is an on-line tool designed for agri- and food and drink businesses, policy makers, farmers, and extension workers to assess their environmental impacts; in particular carbon, biodiversity and water. The tool has been widely used globally (Aryal et al., 2015; Hillier et al., 2012) and previously used to quantify greenhouse gas emissions associated with food items in India (Vetter et al., 2017).

CFTW calculates crop water use based on the FAO56 approach considering atmospheric forcing using the Penman-Monteith equation, crop phenology and crop water stress (Allen et al., 1998).

The Penman-Monteith equation determines reference evapotranspiration *ET*_0_ based on a short well-watered grass considering net radiation, temperature, vapour pressure deficit and wind speed. *ET*_*pot*_ (Eq. (4)) results from the crop specific and climate adjusted crop coefficient *K*_*c*_ and *ET*_0_.

(4) $${\mathrm{ET}}_{\mathrm{pot}}={\mathrm{ET}}_{0}*K_{c}\;$$

Finally the soil water balance is determined based on precipitation, interception, soil water holding capacity, runoff and deep-percolation. The soil water depletion is then used to define crop water stress *K*_*s*_ and *ET*_*a*_ (Eq. (5)).

(5) $${\mathrm{ET}}_{a}={\mathrm{ET}}_{\mathrm{pot}}*K_{s}$$

If an area is irrigated it is assumed that no water stress occurs throughout the growing season (*K*_*s*_ = 1).

The tool employs a global daily climate dataset based on ERA-Interim data (Dee et al., 2011), in addition to the Harmonized World Soil Database (FAO et al., 2012) and crop parameters based on Allen et al. (1998). For this study, ERA-Interim precipitation was replaced with the remote sensing precipitation dataset TRMM (Tropical Rainfall Measurement Mission) (Huffman et al., 2007), which has a finer spatial resolution. Further details about CFTW are provided at Cool Farm Alliance (2016) and Kayatz et al. (2019).

The blue and green water footprint were quantified following the approach of Mekonnen and Hoekstra (2010) and Mekonnen and Hoekstra (2011). Blue and green water use, *CWU*_*green*_ and *CWU*_*blue*_, respectively, were evaluated as follows:

(6) $${\mathrm{CWU}}_{\mathrm{green}}={\mathrm{ET}}_{a}*A$$

(7) $${\mathrm{CWU}}_{\mathrm{blue}}=\left({\mathrm{ET}}_{\mathrm{pot}}-{\mathrm{ET}}_{a}\right)*A_{I}$$

where *A* is the total area under production in one grid cell, district, state or the whole of India, while *A*_*I*_ only refers to the irrigated area for the same spatial unit.

The results were then used to determine the water footprint *WFP* based on production, *P* (Eqs. (8), (9)).

(8) $${\mathrm{WFP}}_{\mathrm{green}}=\frac{{\mathrm{CWU}}_{\mathrm{green}}}{P}$$

(9) $${\mathrm{WFP}}_{\mathrm{blue}}=\frac{{\mathrm{CWU}}_{\mathrm{blue}}}{P}$$

### 2.3 Analysis linking cereal production and water use

This study quantifies the change in cereal production and water use between 2005 and 2014, and evaluates trends in the total area, production, and water use of the five Indian cereals by fitting linear regression models over the ten-year period allowing for direction and strength of change to be observed. Next, the seasonal differences in water footprint and production statistics for each cereal are analysed. All seasonal analysis is assessed through aggregating to monsoon driven seasons (*Kharif*, Autumn and Winter, summarized under *Kharif*) and irrigation dependent seasons (*Rabi* and Summer, summarized under *Rabi*). Wheat is only grown in the *Rabi* season hence omitted from *Kharif* water footprint analysis. Factors associated with water footprints, namely yield and evapotranspiration, are explored and assessed for their importance in explaining the variability of water footprint using the Pearson Correlation Coefficient.

Finally, this study investigates the spatial variability of the improvements in water footprints in the six administrative regions of India (Central India, East India, North India, Northeast India, South India and Western India) to demonstrate if particular regions are driving the observations at national level. Total water footprints are combined per year for each region, and rate of change is determined through linear regression of the regional water footprints over the ten years.

All data processing and analysis of the results was carried out using R software.

## 3 Results

### 3.1 Total area, production and water use between 2005 and 2014

**Fig. 1:**
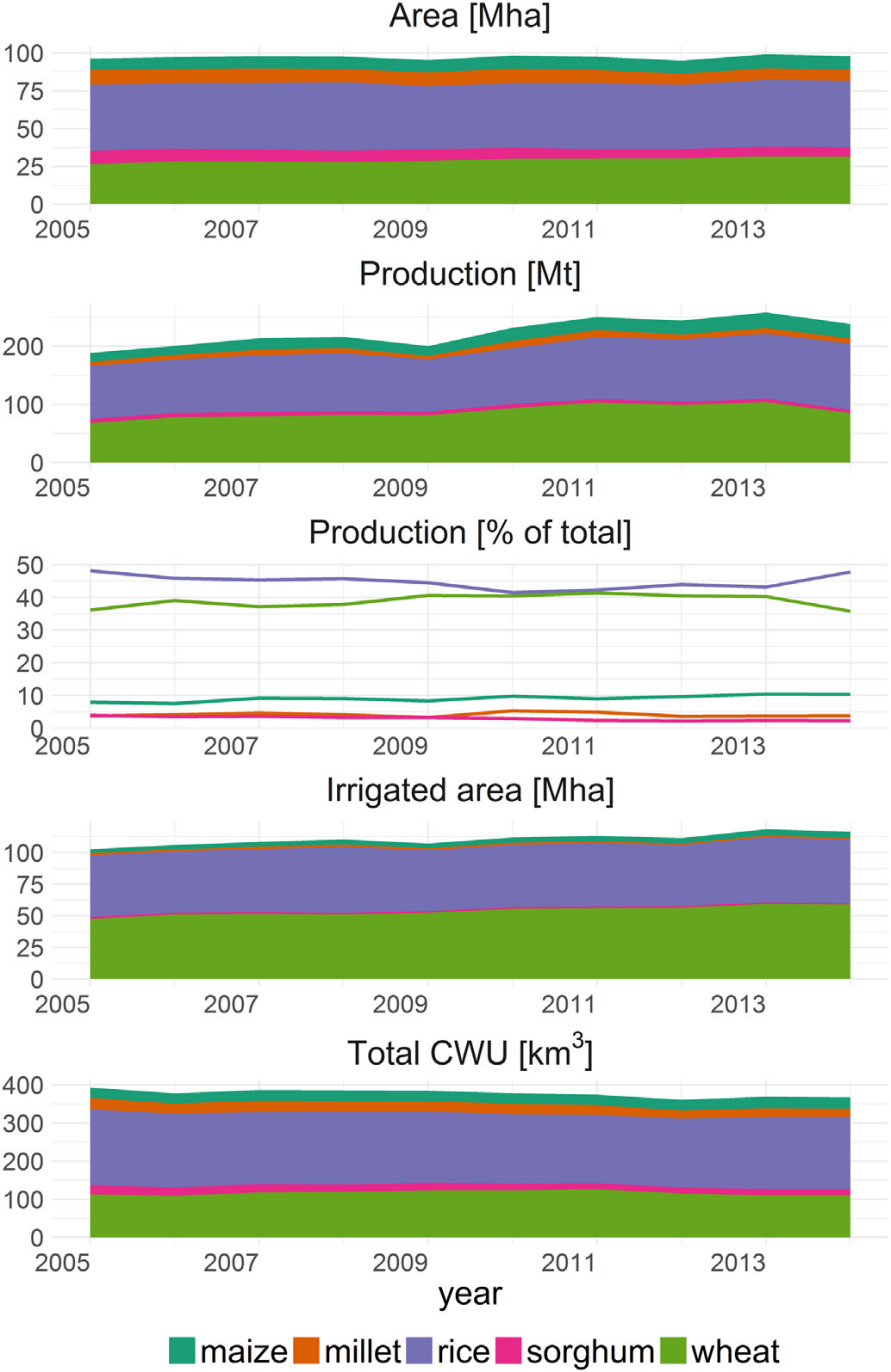
Harvested area, irrigated area, total production and overall water use for maize, millet, rice, sorghum and wheat in India.

Between 2005 and 2014, the harvested land area for cereal production in India increased slightly (+1.8%) from 96.3 to 98.0 Mha and the irrigated land area increased from 51.4 to 58.2 Mha (+13.4%). Total annual cereal production increased by 26.4% from 188.2 Mt to 237.9 Mt (Fig. 1). The average annual total water consumption for cereal production was 377.9 km^3^ over the period 2005–2014 and decreased from 393.2 to 367.1 (−6.6%). Wheat and rice production consumed the greatest amount of water (80.6% of total water use) and the highest consuming states Uttar Pradesh, Punjab and Rajasthan (all in North India), accounted for 20.0%, 8.4% and 8.4% of total Indian water consumption for cereal production, respectively.

The increase in cereal production was not consistent across cereals, with substantial variability evident in respect of absolute and relative annual changes (Table 2, Fig. 1). Maize showed the greatest relative increase in production, while sorghum production marginally decreased over the period. Irrigation area and crop water use increased for both wheat and maize, but decreased for the other cereals.

**Table 2:** Average annual change for area, irrigated area, production and water use based on linear regression models for all values between 2005 and 2014. The number in brackets identifies the percentage change per year based on year 2005.

	Maize	Millet	Rice	Sorghum	Wheat	Total
Area [Mha yr^−1^] ([% yr^−1^])	0.166⁎ (2.10)	−0.263⁎ (−2.78)	−0.039 (−0.09)	−0.290⁎ (−3.42)	0.519⁎ (1.83)	0.094 (0.10)
Irrigated area [Mha yr^−1^] ([% yr^−1^])	0.091⁎ (5.17)	−0.029⁎ (−3.29)	0.063 (0.26)	−0.020⁎ (−2.84)	0.619⁎ (2.43)	0.724⁎ (1.37)
Production [Mt yr^−1^] ([% yr^−1^])	1.229⁎ (8.15)	0.243 (2.95)	2.562⁎ (2.79)	−0.229⁎ (−3.27)	3.074⁎ (3.94)	6.878⁎ (3.44)
Crop water use [km^3^ yr^−1^] ([% yr^−1^])	0.255⁎ (0.95)	−0.947⁎ (−3.52)	−1.180 (−0.61)	−0.913⁎ (−4.15)	−0.040 (−0.04)	−2.825⁎ (−0.75)

⁎ Indicates linear regressions where R^2^ > 0.5 and *p*-value ≤ 0.05.

### 3.2 Drivers of improved cereal water use in India

To identify reasons for increased cereal production with little change in total water use, temporal and spatial variations in cereal water footprints and production were analysed. First, the average yearly and seasonal water footprints were investigated and linked to changes in cereal production and climate. Secondly, the spatial heterogeneity was examined to understand if the improvements occurred in few regions or were ubiquitous.

#### 3.2.1 Differences in seasonal water footprints and cropping patterns

**Fig. 2:**
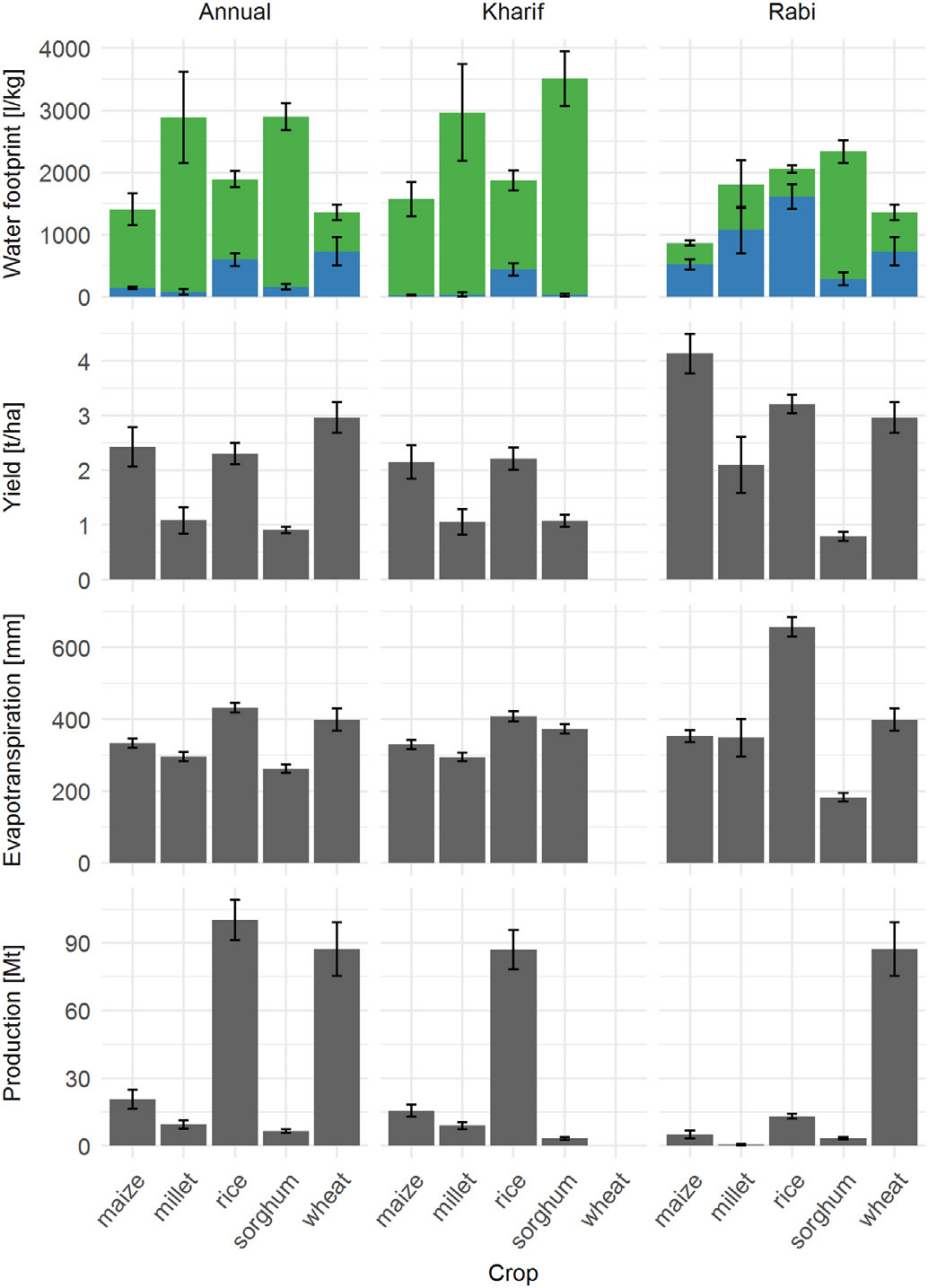
India average blue and green water footprints, yield, evapotranspiration and total production for the years 2005 to 2014 for the whole of India. The error bars indicate the standard deviation of the mean of the 10 years. Wheat is not grown in the Kharif season.

The annual total water footprints of wheat and maize are similar, and only slightly lower than that for rice. Sorghum and millet consistently had the lowest yields across seasons, and the highest annual water footprints of 2894 l kg^−1^ and 2884 l kg^−1^, respectively (Fig. 2).

Water footprints varied substantially by season. Rice has the highest water footprint in *Rabi* followed by sorghum. Rice is the only cereal to have a greater water footprint in *Rab*i than *Kharif:* greater by 10.1%. This is different from all the other cereals whose water footprints were between 45.0% (maize) and 33.4% (sorghum) lower in *Rabi* than *Kharif.*

Cereal blue water footprints during the *Kharif* season were generally small (<1.5% of total water footprint, except for rice). In contrast, cereals blue water footprints during the *Rabi* season ranged from 12.3% of total water footprint for sorghum to 78.3% for rice. Although sorghum and millet had the highest overall water footprint, they were also the crops with lowest blue water footprint.

**Fig. 3:**
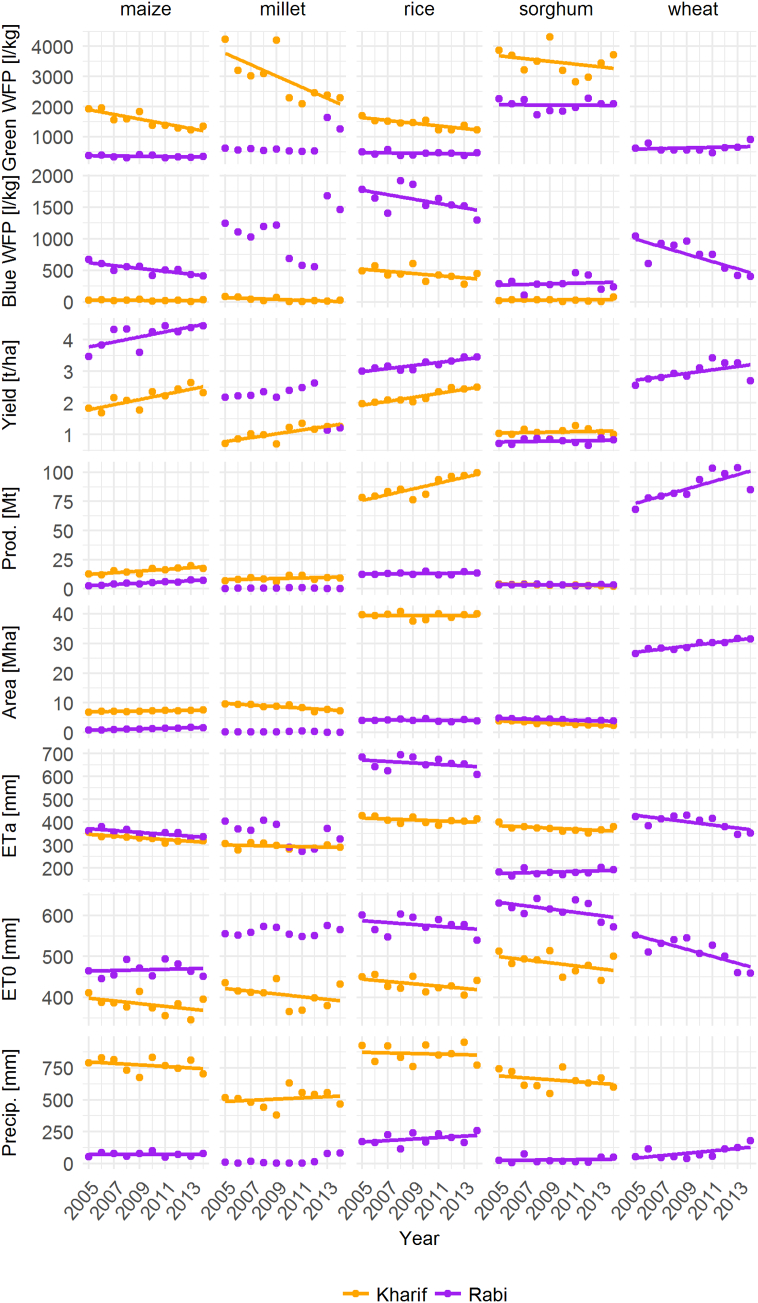
Changes in water footprints, yields, production, area under production, evapotranspiration and precipitation in India, 2005–2014. Lines indicate best fit using a linear regression.

The increase in total cereal production in India over the period 2005–2014 was dominated by rises in *Kharif* production of rice and *Rabi* production of wheat (Fig. 3). The area under cereal production during *Kharif* decreased by 3.0 Mha, shifting to greater cereal production area in *Rabi* that was mainly driven by wheat (+4.9 Mha) and maize (+0.9 Mha).

Total annual water footprints for all cereals also declined over the period 2005–2014, coinciding with an increase in yields and a decrease in evapotranspiration. The reduction of evapotranspiration over time was also apparent for reference evapotranspiration and particular pronounced for wheat. The largest decrease in total water footprints was for millet from 4184 l kg^−1^ to 2324 l kg^−1^ (−44%), corresponding with the largest increase in yield from 0.7 t ha^−1^ to 1.3 t ha^−1^.

In line with changes in total annual water footprints and yields, *Kharif* water footprints have decreased over the study period, especially for green water, while trends in *Rabi* water footprint have been more variable (Fig. 3). Differences in total water footprints in both seasons were strongly correlated with yields (ranging from ρ = −0.78 to ρ = −0.98) (Correlation coefficients are displayed in Table A.1 of the Appendix). The correlation between total water footprints and evapotranspiration was more variable in *Kharif* and *Rabi* (ρ = 0.07 to ρ = 0.89). In particular during *Kharif*, there was strong evidence that green water footprints were positively correlated with evapotranspiration. In *Rabi*, the blue water footprints were associated with evapotranspiration for most crops however relationships were highly variable (ranging from ρ = −0.62 to ρ = 0.95).

#### 3.2.2 Spatial variability of cereal water footprints

**Fig. 4:**
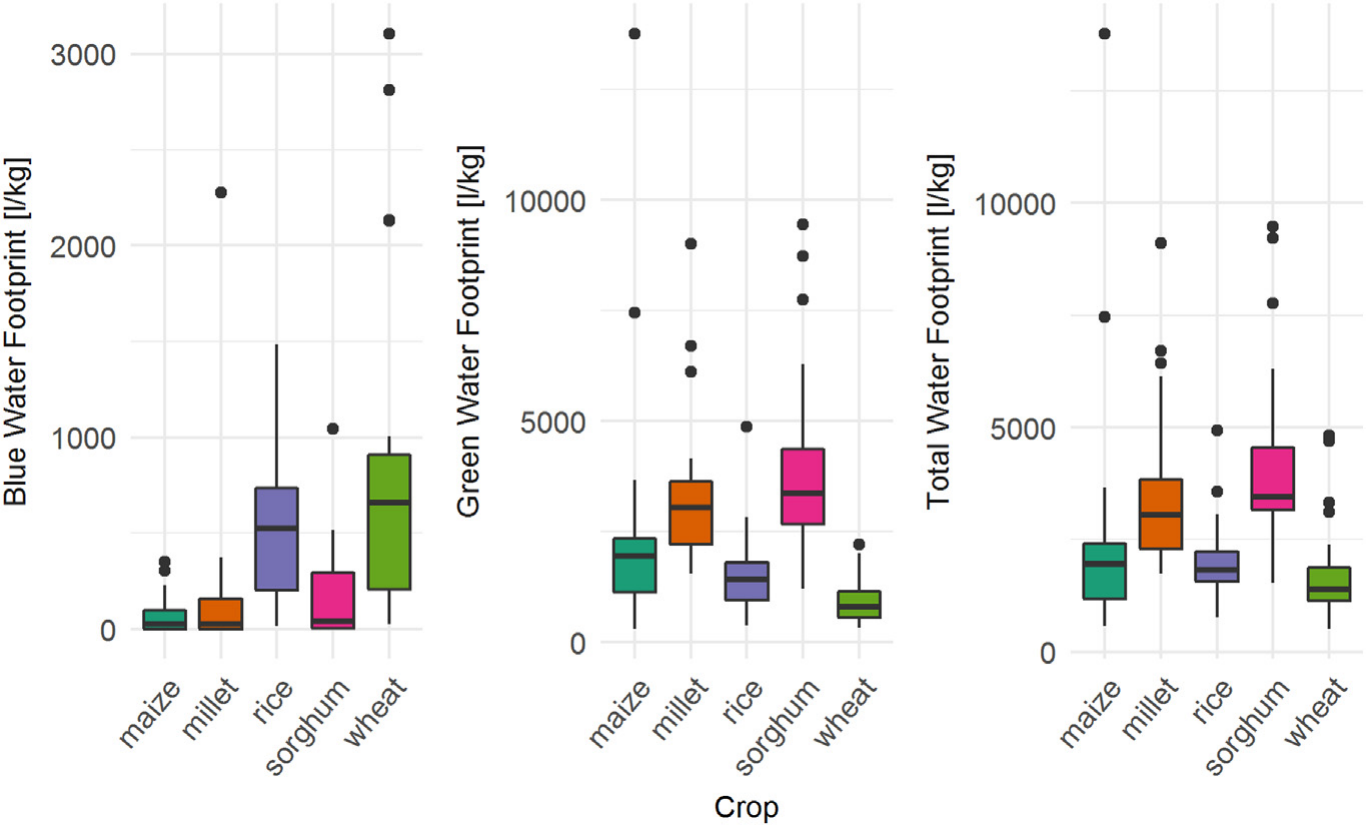
Boxplots of average state-level blue, green and total water footprints between 2005 and 2014.

Cereal water footprints varied markedly between Indian states (Figs. 4, A.1, Table A.2). Rice had the most similar total water footprint across all states but a variable blue water footprint compared to the other cereals. The variation in blue water footprint was even more evident for wheat, which at the same time showed the lowest variability for green water footprints. Total water footprints for wheat were particularly high in the south-eastern states. Maize showed a north-south gradient, with higher water footprints in the northern states. The most heterogeneous total and green water footprints were identified for millet and sorghum, with some states showing sorghum green water footprints as high as 9456 l kg^−1^ (West Bengal) and as low as 1211 l kg^−1^ (Andhra Pradesh). This range was only exceeded by maize due to the high water footprint in Kerala, a state that does not contribute significantly to overall maize production in India. State specific average water footprints and maps of green, blue and total water footprints are provided in the Appendix (Fig. A.1, Table A.2).

**Fig. 5:**
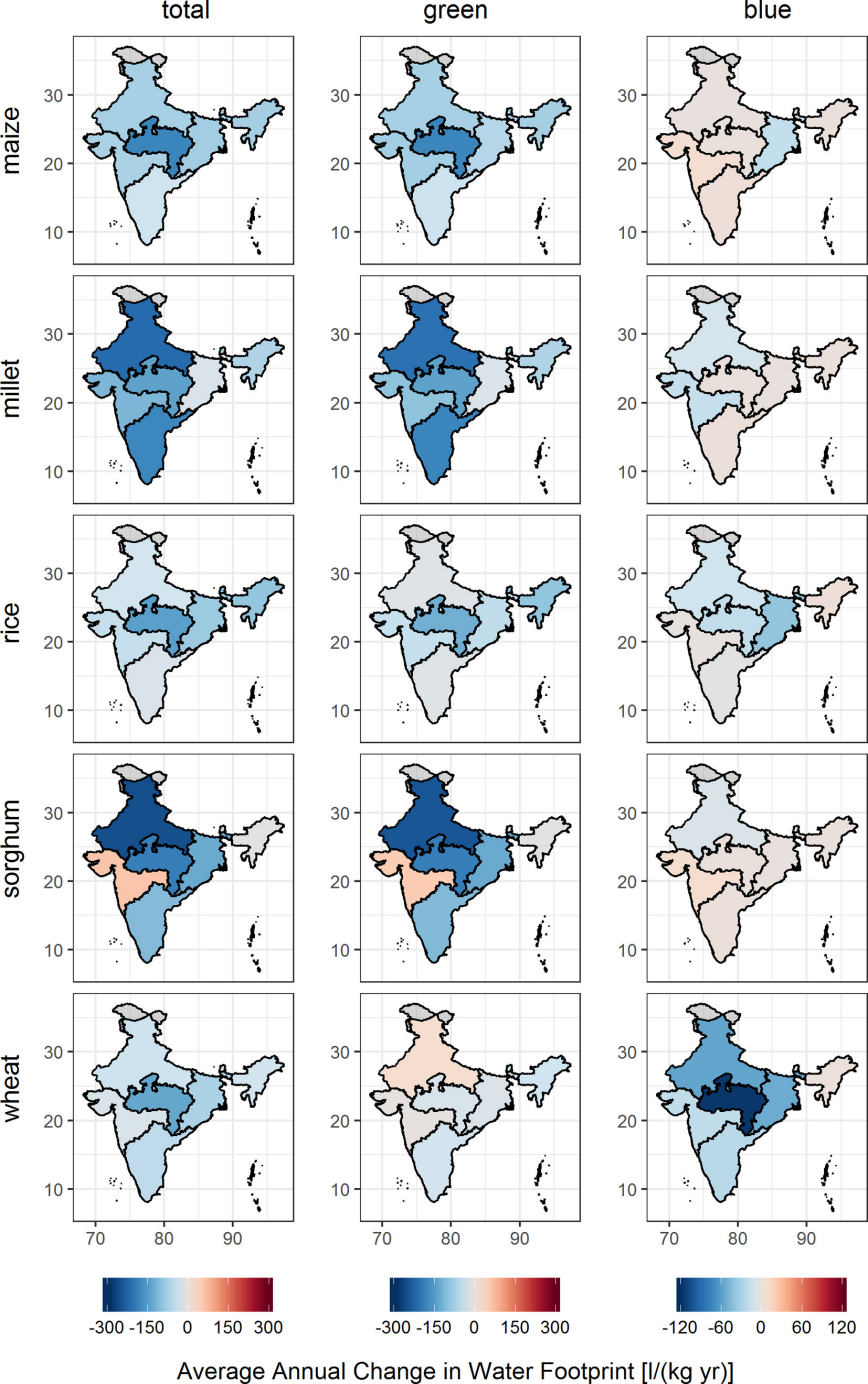
Average change of water footprints per year between 2005 and 2014 for the six administrative divisions in India.

Rate and direction of change in annual water footprints differed in the 6 administrative divisions of India (Fig. 5). There was a decrease in total water footprints in India across almost all administrative divisions and crops. In Central India, the rate of change for total water footprints exceeded all other regions for maize, rice and wheat. The most pronounced changes were simulated for sorghum and millet, where North India showed the highest decreases of 261 l kg^−1^ yr^−1^ and 226 l kg^−1^ yr^−1^ respectively. The annual changes in green water footprints were similar to those seen for the total water footprints, except for wheat where the blue water footprint heavily influences the total. Only wheat had a pronounced change in blue water footprint, decreasing by up to 116 l kg^−1^ yr^−1^ in Central India. This decrease was associated with an increase in yields in this division by 88% and a decrease in evapotranspiration by 10% between 2005 and 2014. Modest increases (not exceeding 52 l kg^−1^ yr^−1^) were identified in blue and green water footprints for individual crops and administrative divisions.

## 4 Discussion

Using Government collated data on cereal production and the CFTW, this study quantified the total water use and water footprints of cereal production over the years 2005–2014. This study shows that over a decade, Indian cereal production increased by 26.4% with relatively small change to water or land used (−6.6% and +1.8%, respectively). This was mainly due to improved cereal yields, which our analysis identifies can in part be explained by a shift towards production in the Rabi season. Substantial differences in water footprints for different cereal crop production were identified: wheat and rice have the lowest green water footprints but the highest blue water footprints, while millet and sorghum were exactly opposite. Maize was the only cereal combining both a low blue and green water footprint. Increasing production of maize but also wheat as a proportion of the total in India has therefore also contributed to a reduction in total water use. This study represents the first regional application of the water component of the CFT.

The reduction in cereal water footprints over the study period was mainly driven by improved yields across India, particularly in the north for sorghum and millet and the central division for rice, maize and wheat. The increase in yield is likely to be largely the result of greater production of high yielding varieties (Ministry of Agriculture and Farmers Welfare, 2017). The increased use of irrigation (51.4 Mha to 58.2 Mha), fertilization (20 Mt to 24 Mt from 2005 to 2014 for cereals) and pesticides (40 kt to 53 kt between 2005 and 2011 for the whole of India) may have also fostered yield increases in India between 2005 and 2014 (Devi et al., 2017; Kumar et al., 2017; Ministry of Agriculture and Farmers Welfare, 2017). However, the increase may also relate to the increased growing area and production during the higher yielding *Rabi* season. This trend has been observed since the 1960's (Davis et al., 2018; Prasanna, 2014). Higher yields during *Rabi* occur for several reasons: temperatures and less cloud cover favour photosynthesis as well as reduce respiration and thus enhance biomass accumulation; improved water management and prevalence of pests (Kailasanathan and Sinha, 1980; Singh et al., 2012). Governmental statistics also suggest that climate related factors are the dominant drivers for lower yields during Kharif, because both, high yielding varieties and traditional varieties, show higher productivity during the dry-season (traditional varieties +36.3%, high yielding varieties +64.2%, average across all cereals based on Ministry of Agriculture and Farmers Welfare, 2017).

As well as a change in yields, this study found a decrease in evapotranspiration that contributed in part to reduced water footprints. The negative trend in evapotranspiration is linked to a trend towards smaller average reference evapotranspiration for the harvest area (see Fig. 3). While this study shows a decrease in reference evapotranspiration for most states and both seasons (>66.7% depending on crop and season), the reduction is most prominent during Rabi (except for Sorghum). The trend in declining evapotranspiration has been reported for 17 out of 19 agroecological regions (Bandyopadhyay et al., 2009) and in various regional studies (Darshana et al., 2013; Jhajharia et al., 2015) in India since the late 1960s. Bandyopadhyay et al. (2009) identified trends in relative humidity (positive trend), wind speed (negative trend) and solar radiation (negative trend) as the drivers of reduced reference evapotranspiration.

Over the study period, the pattern of crop type also slightly shifted in India, with an increase in maize production being most pronounced. Changing crop type has been proposed to reduce water use (Davis et al., 2018). Although yields greatly influence total water footprints, the type of water use is affected by their dominant growing season and irrigation management (Ministry of Agriculture, 2015). Sorghum and millet combine a low yield during *Kharif* season with a low irrigation water use therefore low blue water footprint. Wheat with the highest blue water footprint is exclusively grown as irrigated *Rabi* crop.

### 4.1 Comparison of water use and water footprints with previous estimates

Previous research has estimated that total agricultural water withdrawal in India reached 688 km^3^ a^−1^ in 2010 (FAO, 2016), and is steadily growing. Assuming that this water is applied equally across all irrigated fields (63% of which is used maize, millet, rice, sorghum and wheat production), approximately 433 km^3^ a^−1^ are withdrawn for cereal crops (Government of India, 2018). This study estimated a total blue water use of 105 km^3^ a^−1^ in 2010, suggesting that 75.7% of water withdrawn is lost during conveyance, or *via* runoff and deep percolation.

In terms of India-specific water footprints, earlier estimates from a global assessment by Mekonnen and Hoekstra (2011) and from Kampman (2007) provide a meaningful comparison. The water footprints from this study have similar between-crop differences to the Indian values from Mekonnen and Hoekstra (2011) and Kampman (2007) (Table A.3), except that Sorghum has much lower water footprints compared the other cereals in the later study. The absolute values for the study from 2011 are higher, with the largest discrepancies for maize and sorghum, where the total water footprints of Mekonnen and Hoekstra (2011) are higher by 68.8% and 100.0%, respectively. Similar differences can be found for Kampman (2007) with an offset of up 93.2% for rice. This is potentially due to differences in the years studied, since yields have increased (1996–2005 for Mekonnen and Hoekstra, 2011; 1997–2001 for Kampman, 2007). Additionally, there were methodological differences as alternative data sources (Government collected, as opposed to FAO national statistics) were used and the implementation of the method to calculate evapotranspiration was different. For example Kampman (2007) used Cropwat and monthly average climate for 160 stations of the CLIMWAT dataset to derive state level water footprints. Similar discrepancies to the data provided in Mekonnen and Hoekstra (2011) have been observed by other studies in different contexts, for example Zhuo et al. (2014) in China. More recently, Davis et al. (2018), assessed water productivity of Indian cereals concurring that rice is the most inefficient blue water user during *Kharif* and sorghum has the highest water footprint during *Rabi*. This study builds on this work by assessing more recent years, and applying the CFTW. Other studies investigate virtual water content or water footprints of the same cereals, but do not discuss India-specific figures (Dalin et al., 2012; Hanasaki et al., 2010; Siebert and Döll, 2010; Tuninetti et al., 2015).

Several studies have reported on spatial variability of crop water footprints globally (Dalin and Conway, 2016; Mekonnen and Hoekstra, 2011, Mekonnen and Hoekstra, 2010; Shrestha et al., 2013; Tuninetti et al., 2015). Here inter- and intra-annual variations in crop water footprints for India between 2005 and 2014 are reported. Only one previous study looked at annual variability and showed differing trends in water footprints for various crops, however it focused on the Yellow River basin, China (Zhuo et al., 2016). Differences between *Kharif* and *Rabi* water footprints for 13 crops for most Indian states have been described in Kampman (2007). Kampman (2007) found consistently higher water footprints during *Kharif* similar to our findings, except for rice for which this study identified slightly higher water footprints during *Rabi*. Seasonal variability in cereal water requirements in India also has been shown by Davis et al. (2018), highlighting that for most cereals, *Rabi* production is more efficient, but demands more blue water. Our study demonstrates a clear trend towards lower water footprints and that there are large differences between seasons in India that need to be taken into consideration when trying to reduce environmental impacts of crop production.

### 4.2 Strengths and limitations of this study and water use estimates

This study is strengthened by the use of governmental statistics at the district (662 districts excluding islands) and state (34 states and territories excluding islands) level. This allowed novel relationships to be assessed such as the effect of intra- and inter-annual variability, and changes in yield, area, production and irrigated area at the crop and state-level. Furthermore, by using the newly developed CFTW it demonstrates the applicability of this tool in assessing important agricultural sustainability issues. However, there are some limitation of the data and methodologies used that must be considered.

Although district-level data was used for most input variables, it was not available for irrigation fraction, and therefore state-level data was downscaled based on irrigation fraction of districts in 2005 (Zhao and Siebert, 2015). Newly available remote sensing irrigation products may help in the future to inform this downscaling approach, or even substitute state-level statistics (Ambika et al., 2016). Additionally, data on growing periods was only available at state-level, and therefore growing periods for every state and year are constant in the current analysis. Growing seasons are fairly constant in India, but changes in the onset and end of the monsoon have a clear impact on the length and timing and thus on water footprints (Krishna Kumar et al., 2004). Future work could use dynamic growing seasons, linked to the monsoon and temperature.

In terms of the water use and water footprint estimations, it was assumed, as in previous publications, that once a field is irrigated, the crop does not suffer from water stress at any time during the growing season (Mekonnen and Hoekstra, 2011; Siebert and Döll, 2010). This simplified assumption may lead to an overestimation of blue water use. In addition, there is a correlation between evapotranspiration and biomass production and thus yield, which the FAO56 approach cannot account for (Taylor et al., 1983). This study also assumes constant crop coefficients in India, hence does not distinguish differences between varieties of the same crop, or between management practices that could affect crop coefficients, and therefore crop water demand (Allen et al., 1998). This could be of particular importance in India, where both traditional and high-yielding hybrid cultivars are used (Ministry of Agriculture and Farmers Welfare, 2016).

Finally, the scope of this study was to assess cereal production only and therefore the results described cannot be generalized for other crops or food groups in India. It is likely that reduced cereal harvested area in *Kharif* was substituted by other crops, as area and production of horticulture has gained greatly in importance between 2005 and 2014 in India, with the area increasing from 18.7 Mha to 23.4 Mha, and production from 182.8 Mt to 281.0 Mt (Ministry of Agriculture and Farmers Welfare, 2017). Hence, although the water use for cereals may not have increased, there may have been an increase in Indian agricultural overall. Other factors in terms of water sustainability have not be considered, for example water availability and sources of irrigation water. Although total consumptive water use is important, the environmental impact is additionally determined by the source of water (surface or groundwater), and whether or not water is readily available.

### 4.3 India water security and policy interventions

The findings of our study are relevant given current concerns for ground water depletion and water security in India, and have potential implications for policies related to sustainable food systems (Rodell et al., 2009; Zaveri et al., 2016).

Improving crop yields is a primary means of reducing water use, while maintaining production. Recent yield improvements in India have in part resulted from a shift in the harvested area for cereals from *Kharif* to *Rabi* seasons. While cereal production during *Rabi* removes dependence on fluctuating monsoon precipitation, it results in an increase in irrigation water use; cereal blue water footprints in *Rabi* were 4–30 times higher than those in *Kharif* season. Future research must consider the growing season as a factor contributing to water sustainability, and a full assessment of all crops involved in this transition is needed. Changes in agricultural production practices are also important in improving yields, including the introduction of high yielding cereal varieties and the increased use of irrigation, fertilizers and pesticides (Devi et al., 2017; Kumar et al., 2017; Birhanu and Sekar, 2016; Ministry of Agriculture and Farmers Welfare, 2017; Yadav et al., 2015). However, increased pesticide and fertilizer use may have intensified other environmental impacts including greenhouse gas emissions, biodiversity loss, and grey water footprints. Consideration is required of trade-offs in policies aiming to improve the sustainability of India's food system.

Farmer-level interventions for sustainable water management should focus on increasing irrigation efficiency. This can be partly done through reducing evaporation and thus non-productive water loss. In this context the use of drip and sprinkler apparatus, rather than flood systems, has the potential to reduce groundwater abstraction by around two-thirds (Fishman et al., 2015). Currently over 95% of crops are irrigated using flood and furrow irrigation, triggering evaporation losses and deep percolation (Frenken, 2012). While deep percolating water remains in the catchment, evaporation is lost and does not contribute to crop production *via* transpiration. Furthermore, mulching can reduce evaporation from the soil surface (Chukalla et al., 2015; Ingman et al., 2015; Wang et al., 2018). Rain water harvesting practices may help preserve *Kharif* precipitation for *Rabi* production and decrease water withdrawals for irrigation (Glendenning et al., 2012). However, rain water harvesting may lead to reduced groundwater recharge, lower water availability for downstream users and could also increase evapotranspiration at the catchment level (Glendenning et al., 2012; Kumar et al., 2008). Applying supplementary irrigation in rainfed agriculture has proven effective in stabilising production during fluctuating precipitation (Rockström and Barron, 2007; Sharma et al., 2008). Changing farmer crop choices may also enhance water sustainability (Davis et al., 2017). A switch in production from rice and wheat to maize, sorghum and millet could substantially reduce blue water requirements for cereal production in India. Millet and sorghum production is being promoted by the Government of India, which is considering including these cereals in the public distribution system (Balani, 2013). Changes to cereal production may lead to changes in habitual dietary consumption patterns in India in the future although impacts on consumption are currently poorly understood.

Future research on cereal water footprints in India should explore scenarios for reducing water use that optimize cereal production and water use (total and blue water) by season. Overlaying water use patterns and water footprints with information on sustainable water availability may further help to inform relevant stakeholders (Fishman et al., 2015; Ridoutt and Pfister, 2010). This study has focussed only on the impact of cereal production on water use, and therefore does not provide a holistic assessment of environmental sustainability. Other factors such as climate change, eutrophication and soil erosion are influenced by agricultural management and should be incorporated (Dalin and Rodríguez-Iturbe, 2016). Individual and household-level factors that influence production practices also need due consideration when defining policy responses.

## 5 Conclusions

India's agricultural system has achieved a substantial increase in cereal production over the period 2005–2014 without consuming more water, through improvements in crop productivity and shifting more production to the *Rabi* season. As this has led to greater irrigation area, this strategy is of only limited use in solving water crises in India while sustaining crop production. Reducing pressure on freshwater resources, alleviating unsustainable groundwater use and securing cereal production for food security requires different solutions based on growing season.

Overall, increasing maize production will help to sustain cereal production while minimizing water use, as it is less dependent on blue water, has high yields and can be cultivated during all growing periods in India. In addition, sorghum and millet can help reduce the dependency on freshwater, but substantial investments in improving yields, for example through high yielding varieties, is crucial to maintain production levels.

The data provided in this study will enable decision makers in government, agriculture and food supply chains in India to understand the potential impact of interventions in crop type, cropping season and farming locations on water productivity and cereal production. The study can also contribute to future projections of *per-capita* water demand in India, a country where diets and agricultural production are undergoing a substantial transition.

## Appendix A Supplementary data

Supplementary material Image 1
