# Supplementary material for: “More crop per drop”: Exploring India's cereal water use since 2005
Source: Sci Total Environ. 2019 Jul 10;673:207–17. doi: 10.1016/j.scitotenv.2019.03.304 (PMC6510970; doi:10.1016/j.scitotenv.2019.03.304)
Supplement: Supplementary file 1 — Supplementary material [file mmc1.docx]

# Appendix

Tab. A. 1: Pearson correlation coefficients (CC) between the water footprints of the different seasons and evaporation (ET) and yield (Y) looking at all states and years. * indicates a p value ≤ 0.05.

| Crop | Vari- able | *Annual* | | | *Kharif* | | | *Rabi* | | |
| --- | --- | --- | --- | --- | --- | --- | --- | --- | --- | --- |
|  |  | Total | Green | Blue | Total | Green | Blue | Total | Green | Blue |
| Maize | CC ET | 0.8* | 0.83* | -0.13 | 0.72* | 0.73* | 0.14 | 0.68* | 0.12 | 0.78* |
|  | CC Y | -0.98* | -0.97* | -0.4 | -0.97* | -0.97* | -0.72* | -0.95* | -0.75* | -0.80* |
| Millet | CC ET | 0.56 | 0.58 | 0.21 | 0.49 | 0.5 | 0.2 | 0.45 | 0.13 | 0.72* |
|  | CC Y | -0.98* | -0.98* | -0.9* | -0.98* | -0.98* | -0.89* | -0.96* | -0.96* | -0.85* |
| Rice | CC ET | 0.77* | 0.65* | 0.66* | 0.69* | 0.5 | 0.63* | 0.89* | -0.47 | 0.93* |
|  | CC Y | -0.96* | -0.87* | -0.75* | -0.95* | -0.89* | -0.55 | -0.92* | -0.02 | -0.81* |
| Sorghum | CC ET | 0.49 | 0.6 | -0.68* | 0.59 | 0.58* | 0.4 | 0.07 | 0.41 | -0.62 |
|  | CC Y | -0.78* | -0.67* | -0.41 | -0.97* | -0.97* | -0.28 | -0.78* | -0.46 | -0.73* |
| Wheat | CC ET | 0.72* | -0.71* | 0.95* |  |  |  | 0.72* | -0.71* | 0.95* |
|  | CC Y | -0.82* | -0.47 | -0.38* |  |  |  | -0.82* | -0.47 | -0.38 |


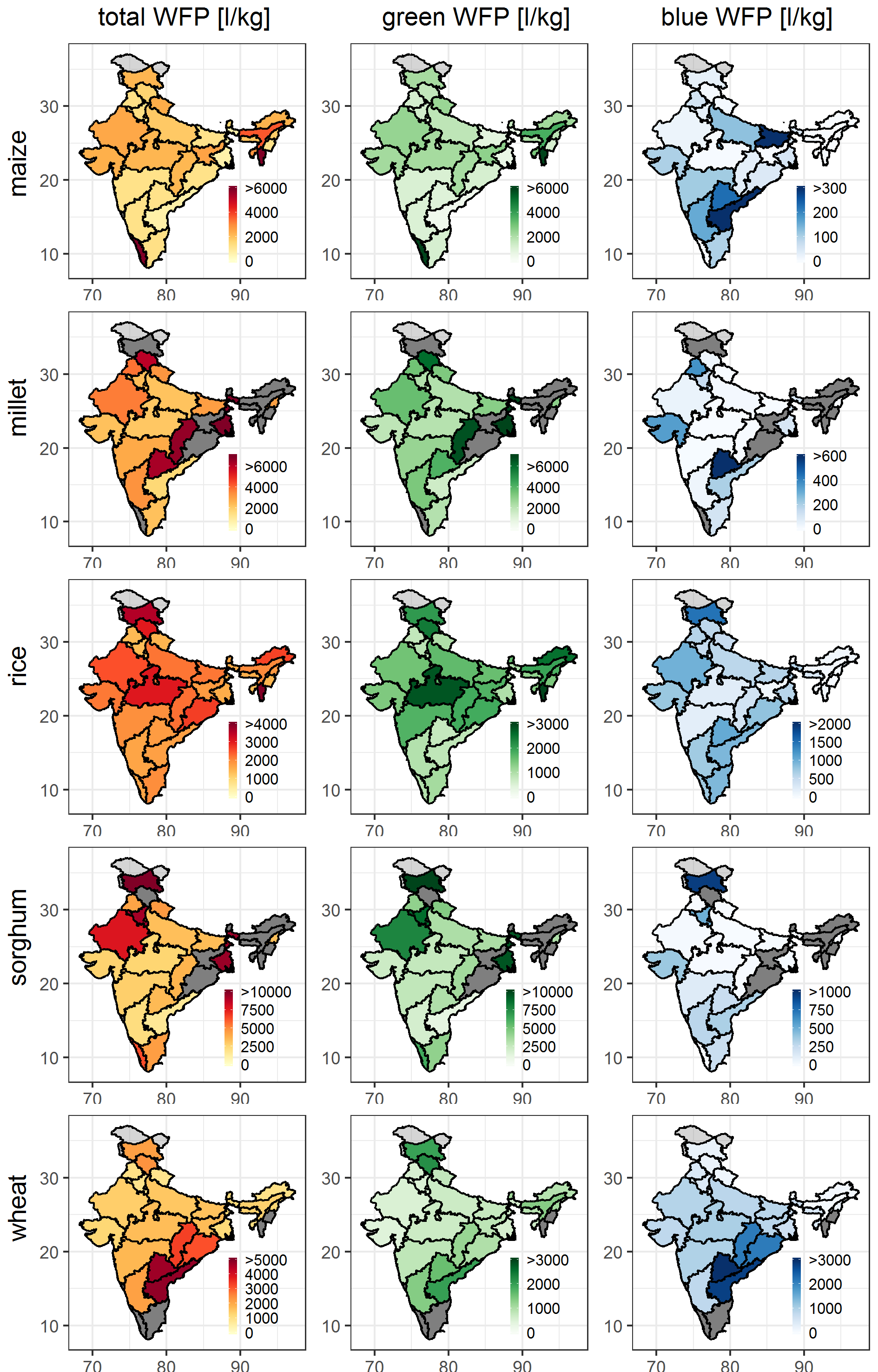


Fig. A. 1: Total, Green, and Blue water footprints at state-level for all of India for maize, millet, rice, sorghum and wheat between 2005 and 2014. Dark grey = no significant production, light grey = disputed area.

Tab. A. 2: Average state-level water footprints for India between 2005 and 2014.

| State | Blue Water Footprint [l/kg] | | | | | Green Water Footprint [l/kg] | | | | | Total Water Footprint [l/kg] | | | | |
| --- | --- | --- | --- | --- | --- | --- | --- | --- | --- | --- | --- | --- | --- | --- | --- |
|  | Maize | Millet | Rice | Sorgh-um | Wheat | Maize | Millet | Rice | Sorgh-um | Wheat | Maize | Millet | Rice | Sorgh-um | Wheat |
| Andhra Pradesh | 307 | 200 | 917 | 332 | 2811 | 300 | 1560 | 832 | 1211 | 2016 | 607 | 1760 | 1748 | 1543 | 4827 |
| Arunachal Pradesh | 0 | - | 58 | - | 63 | 2234 | - | 2572 | - | 926 | 2234 | - | 2630 | - | 989 |
| Assam | 0 | - | 40 | - | 28 | 3665 | - | 1983 | - | 1349 | 3665 | - | 2023 | - | 1376 |
| Bihar | 349 | 2 | 611 | 10 | 869 | 800 | 3103 | 1585 | 3403 | 756 | 1149 | 3105 | 2196 | 3414 | 1624 |
| Chandigarh | 99 | - | 386 | - | 80 | 2047 | - | 386 | - | 433 | 2146 | - | 772 | - | 513 |
| Chhattisgarh | 28 | 0 | 381 | 5 | 2131 | 2108 | 6706 | 1869 | 3680 | 1203 | 2137 | 6706 | 2250 | 3685 | 3334 |
| Dadra and Nagar Haveli | 0 | - | 692 | 0 | 908 | 3095 | - | 1585 | 3956 | 728 | 3095 | - | 2277 | 3956 | 1636 |
| Daman and Diu | - | - | 300 | 517 | 689 | - | - | 1385 | 2778 | 471 | - | - | 1685 | 3295 | 1160 |
| Goa | - | - | 619 | - | - | - | - | 971 | - | - | - | - | 1590 | - | - |
| Gujarat | 99 | 343 | 769 | 379 | 813 | 2196 | 1952 | 1409 | 2310 | 449 | 2295 | 2295 | 2178 | 2689 | 1263 |
| Haryana | 25 | 143 | 488 | 493 | 443 | 1250 | 2231 | 885 | 8727 | 499 | 1275 | 2374 | 1374 | 9219 | 942 |
| Himachal Pradesh | 5 | 30 | 559 | - | 185 | 1592 | 6116 | 2461 | - | 2212 | 1596 | 6146 | 3021 | - | 2396 |
| Jammu and Kashmir | 26 | - | 1485 | - | 213 | 2140 | - | 2090 | - | 1978 | 2166 | - | 3576 | - | 2191 |
| Jharkhand | 2 | - | 62 | - | 862 | 2553 | - | 1766 | - | 846 | 2554 | - | 1828 | - | 1709 |
| Karnataka | 155 | 26 | 731 | 284 | 815 | 1004 | 3348 | 946 | 1961 | 1360 | 1160 | 3374 | 1677 | 2245 | 2175 |
| Kerala | 0 | - | 734 | 7 | - | 13772 | - | 939 | 6295 | - | 13772 | - | 1673 | 6302 | - |
| Madhya Pradesh | 2 | 0 | 227 | 4 | 1008 | 2162 | 2155 | 2841 | 2602 | 689 | 2165 | 2155 | 3068 | 2606 | 1697 |
| Maharashtra | 109 | 2 | 196 | 127 | 956 | 1049 | 2814 | 1765 | 2689 | 830 | 1158 | 2816 | 1960 | 2817 | 1786 |
| Manipur | 0 | - | 14 | - | - | 1159 | - | 1440 | - | - | 1159 | - | 1454 | - | - |
| Meghalaya | 0 | - | 290 | - | 463 | 1958 | - | 1277 | - | 910 | 1958 | - | 1567 | - | 1373 |
| Mizoram | 4 | - | 59 | - | - | 7453 | - | 4881 | - | - | 7458 | - | 4940 | - | - |
| Nagaland | 1 | 0 | 42 | 0 | 180 | 1746 | 3050 | 1807 | 3302 | 1104 | 1748 | 3050 | 1849 | 3302 | 1283 |
| NCT of Delhi | 137 | 3 | 438 | 63 | 337 | 2312 | 2308 | 576 | 4830 | 341 | 2449 | 2311 | 1014 | 4893 | 678 |
| Odisha | 41 | - | 813 | - | 2133 | 1118 | - | 1871 | - | 982 | 1159 | - | 2684 | - | 3114 |
| Puducherry | - | 241 | 1395 | 1046 | - | - | 1600 | 672 | 2267 | - | - | 1842 | 2067 | 3313 | - |
| Punjab | 54 | 372 | 599 | 0 | 445 | 1170 | 3548 | 803 | 4217 | 567 | 1224 | 3920 | 1402 | 4217 | 1012 |
| Rajasthan | 17 | 38 | 975 | 14 | 914 | 2435 | 3715 | 1516 | 7756 | 511 | 2452 | 3753 | 2491 | 7770 | 1425 |
| Sikkim | 0 | - | 24 | - | 55 | 1527 | - | 1629 | - | 1135 | 1527 | - | 1654 | - | 1190 |
| Tamil Nadu | 97 | 110 | 909 | 228 | - | 1123 | 2180 | 1082 | 4218 | - | 1220 | 2290 | 1991 | 4446 | - |
| Telangana | 229 | 2277 | 1039 | 237 | 3107 | 919 | 4157 | 767 | 3249 | 1582 | 1148 | 6434 | 1806 | 3486 | 4689 |
| Tripura | 0 | - | 29 | - | 421 | 2902 | - | 1345 | - | 678 | 2903 | - | 1374 | - | 1099 |
| Uttar Pradesh | 124 | 13 | 563 | 1 | 855 | 1686 | 2230 | 1649 | 3345 | 690 | 1810 | 2243 | 2212 | 3346 | 1545 |
| Uttarakhand | 6 | 0 | 477 | - | 123 | 2365 | 3295 | 982 | - | 787 | 2371 | 3295 | 1459 | - | 909 |
| West Bengal | 49 | 94 | 589 | 17 | 630 | 536 | 9011 | 977 | 9456 | 569 | 586 | 9105 | 1567 | 9473 | 1200 |

Tab. A. 3: Comparison of green, blue and total water footprints between this and other studies. The results of this study give the average of all years and also the respective standard deviation.

| Crop | This study | Mekonnen and Hoekstra (2011) (Differences) | Difference between studies |
| --- | --- | --- | --- |
| Maize | 1404 ± 259 1264 ± 254 140 ± 19 | 2342 (+66.8%) 2239 (+77.1%) 103 (-26.4%) | 2171 (+54.6%) 1989 (+57.4%) 182 (+30.0%) |
| Millet | 2884 ± 773 2805 ± 734 79 ± 44 | 3795 (+31.6%) 3719 (+32.6%) 76 (-3.8%) | 3948 (+36.9%) 3892 (+38.8%) 56 (-29.1%) |
| Rice | 1892 ± 199 1296 ± 131 596 ± 103 | 1836 (-3.0%) 1394 (+7.6%) 452 (-24.2%) | 3655 (+93.2%) 2692 (+107.7%) 963 (+61.6%) |
| Sorghum | 2895 ± 212 2736 ± 218 158 ± 42 | 5792 (+100.1%) 5694 (+108.1%) 98 (-38.0%) | 3219 (+11.2%) 2918 (+6.7%) 301 (+90.5%) |
| Wheat | 1359 ± 181 630 ± 127 729 ± 231 | 1805 (+32.8%) 634 (0.6%) 1171 (60.6%) | 1036 (-23.8%) 214 (-66.0%) 822 (+12.8%) |
